# Supplementary material for: Bromine Ion-Intercalated Layered Bi2WO6 as an Efficient Catalyst for Advanced Oxidation Processes in Tetracycline Pollutant Degradation Reaction
Source: Nanomaterials (Basel). 2023 Sep 21;13(18):2614. doi: 10.3390/nano13182614 (PMC10537847; doi:10.3390/nano13182614)
Supplement: Supplementary file 1 [file nanomaterials-13-02614-s001.zip › nanomaterials-2617715-supplementary conversion.pdf]

Bromine Ion-Intercalated Layered Bi_2WO_6 as an Efficient Catalyst for Advanced Oxidation Processes in Tetracycline Pollutant Degradation Reaction

Rama Krishna Chava * and Misook Kang *

Department of Chemistry, College of Natural Sciences, Yeungnam University, 280 Daehak-ro, Gyeongsan, Gyeongbuk 38541, Republic of Korea

* Correspondence: drcrkphysics@hotmail.com or rama@ynu.ac.kr (R.K.C.); mskang@ynu.ac.kr (M.K.)

Visible photocatalytic tetracycline degradation experiments of Bi_2WO_6 nanostructures

The corresponding aqueous TC absorbance spectra (monitored at 358 nm) of BW samples at different stages of the light irradiation were provided in Fig. 10a-d. The absorption intensity of TC at 358 nm declines gradually with the irradiation time, proposing that TC molecules in an aqueous solution are destructed through the visible-light-driven photocatalytic reaction.

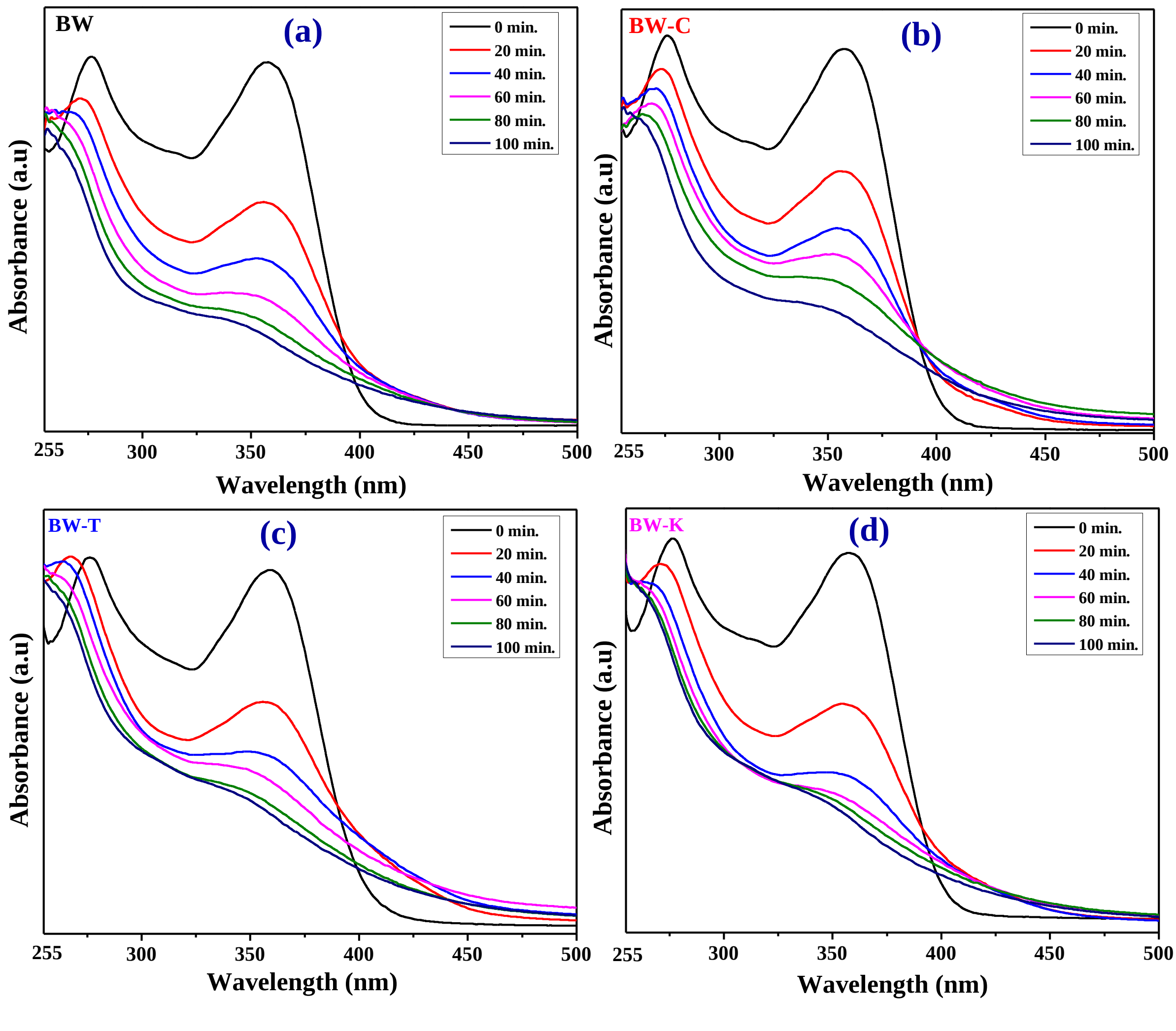

Figure S1. Visible light driven tetracycline degradation curves over (a) BW, (b) BW-C, (c) BW-T and (d) BW-K photocatalysts.

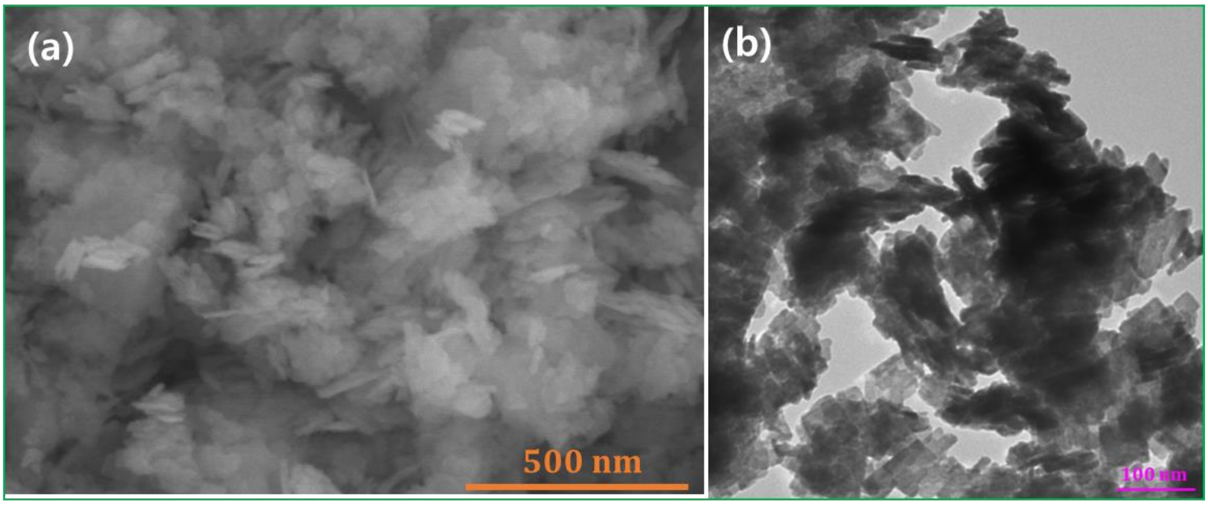

Figure S2. (a) FE-SEM and (b) TEM images of the BW-K photocatalyst sample after cycling reactions.

Table S1. Bi_2WO_6 based photocatalysts for pollutant degradation reactions

Photocatalyst	Light source	Pollutants	Photocatalytic efficiency	Reference
ultrathin Bi_2WO_6 nanosheet	300 W Xe lamp λ>420 nm	sulfamethoxazole	63%	Ma, et al., 2023[32]
3D flower-like Bi_2WO_6	300 W Xe lamp λ>420 nm	Tetracycline	49%	Li, et al., 2023[39]
$BiOBr/Bi_2WO_6$	1000 W Xe lamp λ>420 nm	Phenol	65%	Pancielejko, et al., 2021[50]
I-doped Bi_2WO_6 Nanosheets	Fenton process	Bisphenol A	78%	Xu, et al., 2021[38]
Bi_2WO_6/DMPBP[5]	350 W Xe lamp λ>420 nm	Rhodamine B	93%	Jia, et al., 2023[24]
Br-terminated 2D Bi_2WO_6	300 W Xe lamp λ>420 nm	Methyl Orange	-	Hu, et al., 2021[59]
$Bi_2W_xMo_{1-x}O_6$	30 W LED	Methylene Blue	56.6%	Belousov, et al, 2023[23]
$Bi_2WO_6/ZnSnO_3$	300 W Xe lamp λ>420 nm	Rhodamine B	98%	Zhao, et al., 2023[53]
WO_3/Bi_2WO_6<br>CdS/ Bi_2WO_6	350 W Xe lamp λ < 420 nm	$K_2Cr_2O_7$<br>Rhodamine B	53.82%<br>60.82%	Zhao, et al., 2023[51]
I-doped Bi_2WO_6	150 Xe lamp λ < 400 nm	Rhodamine B	80 %	Wang, et al., 2018[48]
C-doped Bi_2WO_6	300 W Xe lamp λ>420 nm	Tetracycline	84.6%	Jiang, et al., 2023[54]
Br^{-} intercalated Bi_2WO_6 nanoplates	150 W Xe lamp λ>420 nm	Tetracycline 20 ppm	84%	Present work

References

[59] Hu, S. J., Fei, Q. R., Li, Y. J., Wang, B. L., Yu, Y. J., 2021. Br-terminated 2D Bi_2WO_6 nanosheets as a sensitive light-regenerated electrochemical sensor for detecting sulfamethoxazole antibiotic. Surf. Interfaces, 25, 101302. https://doi.org/10.1016/j.surfin.2021.101302
